# Effects of monosodium glutamate on apoptosis of germ cells in testicular tissue of adult rat: An experimental study

**DOI:** 10.18502/ijrm.v17i4.4551

**Published:** 2019-06-13

**Authors:** Fatemeh Rahimi Anbarkeh, Raheleh Baradaran, Nasibeh Ghandy, Mehdi Jalali, Mohammad Reza Nikravesh, Mohammad Soukhtanloo

**Affiliations:** ^1^ Department of Anatomy and Cell Biology, Faculty of Medicine, Mashhad University of Medical Sciences, Mashhad, Iran.; ^2^ Department of Clinical Biochemistry, Faculty of Medicine, Mashhad University of Medical Sciences, Mashhad, Iran.

**Keywords:** *Apoptosis*, * Monosodium glutamate*, * Rat*, * Testis*, * Vitamin C*

## Abstract

**Background:**

Monosodium glutamate (MSG) is used as a flavoring and food seasoning. Some studies have reported the oxidative effects of using this substance on various tissues.

**Objective:**

This study has investigated the effects of MSG and the protective effect of vitamin C (vit C) on apoptosis of testicular germ cells and biochemical factors.

**Materials and Methods:**

In this experimental study, 24 adult male Wistar rats were randomly divided into four groups: control (received distilled water), vit C group (150 mg/kg), experimental group 1 (MSG 3 gr/kg), experimental group 2 (MSG 3 gr/kg + vit C 150 mg/kg). The rats were gavaged for 30 days, and then were sacrificed, the right testis was isolated for biochemical examinations for the glutathione, malondialdehyde, and left testis used in histological experiments. Tunnel staining was used to determine the number of apoptotic cells.

**Results:**

The results showed that apoptotic cells in the MSG group had a significant increase compared to the control group (P = 0.001), but the number of these cells in the MSG co-administered with vit C and vit C groups were significantly lower than the MSG group. Germinal epithelial thickness also decreased in MSG group compared to the control group.

**Conclusion:**

MSG can lead to increase apoptotic changes in the germinal epithelial of the testicle, and vit C as an antioxidant can modify the pathological and biochemical changes induced by MSG.

## 1. Introduction

Glutamate is known as current amino acids in nature that organize an important part of many peptides and proteins in most tissues. It is produced in the body and acts the main role in the metabolism of the body (1). Monosodium glutamate (MSG) is used as an additive for maintenance of food indices, such as the quality, taste, and durability. MSG is prepared from starch, sugar, beet, sugar cane, or molasses (2). It is used as a seasoning and flavoring in many countries. It may be used in packaged foods without mentioning the label 2. There is a controversy over the use of MSG in foods worldwide (3).

MSG can induce oxidative stress with the production of oxygen radicals and hydrogen peroxide, leading to oxidative DNA damage and peroxidation of cell membranes and cell death. Internal antioxidants including vitamins, minerals may inhibit reactive oxygen species or peroxidation of lipids (4).

Several studies have reported the toxic effects of MSG on human and animal tissues. MSG can cause to change in kidney function, liver, and lipid profile (5–7). Histological findings in the ovaries have demonstrated evidence of cellular hypertrophy, degenerative and atrophic changes that may be the cause of infertility in women (8).

Testicular tissue is one of the most sensitive tissues and can be affected by environmental risk factors (9). Infertility, testicular hemorrhage, morphological changes, and sperm production have been reported after the administration of MSG in male rats (10). Studies have shown the effect of MSG on testicular tissue and the overall structure by conventional staining but have not assessed the amount of apoptotic germinal epithelial cells yet.

The aim of this study was to evaluate the effect of MSG on testicular germ cell apoptosis and biochemical changes in testicular tissue as well as the protective effect of vitamin C (vit C) in concomitant administration with MSG.

## 2. Materials and Methods

### Animals

To this study, we purchased 24 mature male Wistar rats (body weight (BW): 200 ± 250 gr, age: 6–8 wk old) from the animal house of the Faculty of Medicine, Mashhad University, Iran. The rats were kept in a well-ventilated room under controlled conditions (22–25°C, 40–70% relative humidity, 12 hr light/darkness cycle) and away from any stressful conditions. They were allowed free access to food pellets and drinking water.

### Chemicals

MSG (C5H8NNaO4 ((Negin Tejarat Payam Co., Iran under the license of Huifenghe, China), Vit C Vials (Osve Co., Iran), and TUNEL kit was purchased from Roche, Germany.

### Experimental design

Animals were randomly allocated into four groups (N = 6) as follows:

Control group received distilled water;

Vit C group received vit C 150 mg/kg/BW/Day;

Experimental group 1 received MSG 3gr/kg/BW/Day; and

Experimental group 2 received MSG 3gr/kg/BW/Day and vit C 150 mg/kg/BW/Day (11, 12).

Treatments were done within 30 days by oral gavage mode. The BW of the rats was recorded weekly at the beginning and end of the experimental duration. The first day, when the animals were treated was considered experimental day 0. At the end of the 30 days of treatment, all animals were anesthetized with chloroform and the abdominal cavity was opened up to expose the testis. The testis tissues after perfusion of heart for full formalin penetration were quickly processed for light microscope investigations and biochemical assessments. The left testes were used for the test of TUNEL and right testes were used for evaluating the level of malondialdehyde (MDA) and glutathione (GSH).

### Histological procedures of the testis

We dissected and weighted left testes quickly for doing histological techniques, fixed in 10% formalin. Afterward, the tissues were dehydrated in an ascending grade of alcohol, cleared in xylene, and embedded in paraffin. Using a rotator microtome, serial sections of 5µ thick were taken. Briefly, tissue sections were deparaffinized by xylene and were hydrated in the descending alcohols. Samples were incubated in hydrogen peroxide diluted with methanol (3% solution). They were then incubated with a solution of proteinase K diluted in PBS for 15 min at room temperature in a moist environment. Tissue sections were incubated with TUNEL solvent solution diluted with 50 ml of specific solvent for 2 h at 37°C in a moist environment. Then samples were incubated with a solution convert (Convert POD, HRP) at a rate of 50 ml for 60 min at 37°C in a moist environment. Tissue sections were incubated with a chromogen solution diaminobenzidine tetrachloride (DAB) for 15 min at room temperature. As a final step, sections were counterstained with hematoxylin for 10 sec, dehydrated in ascending alcohols, and were cleared with xylene. Apoptotic cells appeared in brown. Images were captured at 200× and 400× magnifications using Olympus light microscope and were transferred to a computer using a high-resolution camera (BX51, Japan). Morphometric methods were used to evaluate TUNEL-positive cells per unit area in testis by two treatment blinded observers. The number of TUNEL-positive cells was counted using grades unbiased frames. "We calculated the mean number of TUNEL-positive cells per unit area (NA) of testis in different groups of rats using the following formula (13): 

$$N=\frac{\Sigma Q}{\left(a/f\right)\Sigma p}$$

In the above formula, “ΣQ” indicates the sum of counted particles appeared in sections, “a/f” indicates the area associated with each frame, and “ΣP” indicates the sum of frame associated points touching space."

### Tissues homogenates and estimation of antioxidant parameters

After washing the testicular tissue and removing fatty parts, the tissues were homogeneous in 50 mµ sodium phosphate buffer (pH: 7.4) containing 0.1 mµ ethylenediaminetetraacetic acids (EDTA) on ice-cold. We separated supernatant after centrifugation at 5000 rpm for 20 min at 40°C. To analyze biochemical parameters, supernatant was used.

### Reduced glutathione (GSH) assay

To determine the thiol groups, with the aid of Hu, Colorimetric method was used from 5, 5'-dithiobis (2-nitrobenzoic acid). DTNB with thiol groups creates a yellow compound that has a maximum optical absorption in 412 nm wavelength and its Optical absorption coefficient is 13.6 Mm${}^{--1}$ cm${}^{--1}$ (14). Enzyme activity was expressed as mmol/gr tissue. For this purpose, 50 μl of homogenates were blended with 1 ml of 10 mM EDTA- Tris buffer and the absorbance was evaluated at 412 nm by using a spectrophotometer (Jenway 6105 UV/vis, UK) (A${}_{1}$). To this solution was mixed 20 µl DTNB (10 mM), and after 10 min at the laboratory temperature, sample absorption was evaluated (A${}_{2}$). Absorption of DTNB solution was evaluated as Blank alone (B). The total amount of thiol groups was calculated using the following formula:

$$A=A{}_{1}-A{}_{2}-B.$$

Total of thiol groups $=A\frac{1.07}{0.05\times 13.6}$

### Measurement of MDA

The amount of MDA was determined in the homogenized tissue samples by the aid of the TBA method. The compound of MDA–TBA can be easily measured in a fluorimetric method (wavelength of irradiation/radiation = 485/535) (15). To prepare a standard curve, 2.39 µl tetraethoxypropane was added to 10 ml of distilled water. This solution was used to prepare dilutions of (3.12, 6.25, 12.5, 25, 50, 100, 200 µmol/ml) in different tubes.

In the start the work, we mixed 0.5 ml homogenated tissue with 0.5 ml of distilled water and 0.5 ml TBA. Then the compound was heated for 60 min in a boiling water bath. As soon as cooling the compound, 25 µl of HCL (PH = 1.6-1.7) and 1.5 ml of n-butanol was added to it and vortex-mixed for 1 min followed by centrifugation at 1000 gr for 10 min. At that point, we separated the upper liquid and transferred to fresh tubes and the absorbance level was read using a fluorimeter (PerkinElmer VICTOR X5 USA). We obtained a calibration curve using MDA tetrabutylammonium. The MDA levels were considered as nmol/gr tissue.

### Ethical consideration

This study was approved by the Animal Ethics Committee of Mashhad University, according to the recommendations of the national guidelines (IR.MUMS.fm.REC.1395.611).

### Statistical analysis

Statistical calculations were performed using SPSS software (Statistical Package for the Social Sciences, version 20.0, SPSS Inc, Chicago, Illinois, USA). Statistical significance between groups was evaluated using one-way ANOVA analysis of variance. Differences between groups were determined by the Tukey test. Results were presented as mean ± SD with p < 0.05 considered to be statistically significant.

## 3. Results

### Effect of MSG and Vit C on BW and testes' weight

Final BWs increased in all of the groups. The comparison of the final BWs with the initial BWs (weight changes) between groups revealed a significant difference in the MSG-treated group with the control group. Likewise, there was a significant difference in the MSG-treated group in comparison with the vit C group (p = 0.021).

A significant difference in the testes' weight of the rats was observed between groups after administration of MSG within a month (Table I).

### Effect of MSG and vit C on GSH and MDA level in testis tissue 

Evaluation MSG plus vit C group with the control group revealed a significant reduction in MDA level. Likewise, we revealed the MSG plus vit C group in compare with the MSG-treated group had a significant decrease in MDA level. This result indicate effective role of vit C in diminishing MDA levels. There was no significant difference in GSH levels between groups after the administration of MSG within a month (p > 0.05) (Table II).

### Histological results

#### Effect of MSG and vit C on TUNEL-positive cell numbers

**Figure 1:**
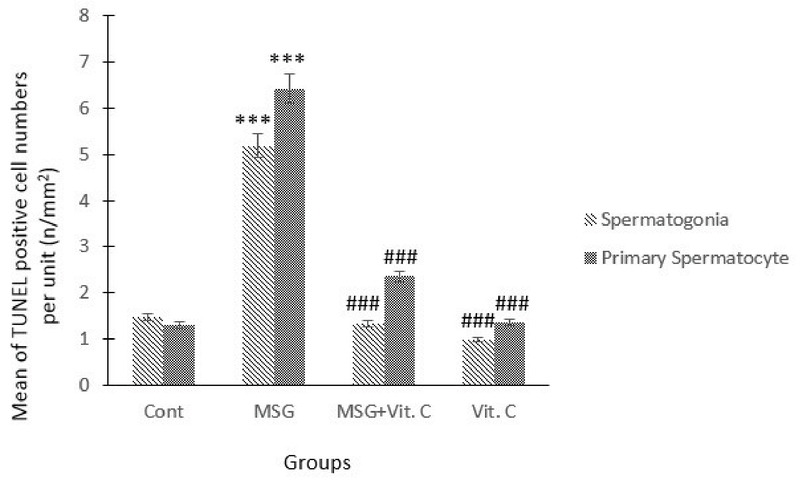
Effects of treatment of MSG, vit C, and their combinations on TUNEL-positive spermatogonia and primary spermatocyte numbers in the testis tissues of rats. Note: ***represents a significant difference between the MSG-treated group and the control group (p < 0.001); ###represents a significant difference in vit C and MSG + vit C group with MSG group (p < 0.001).

**Figure 2:**
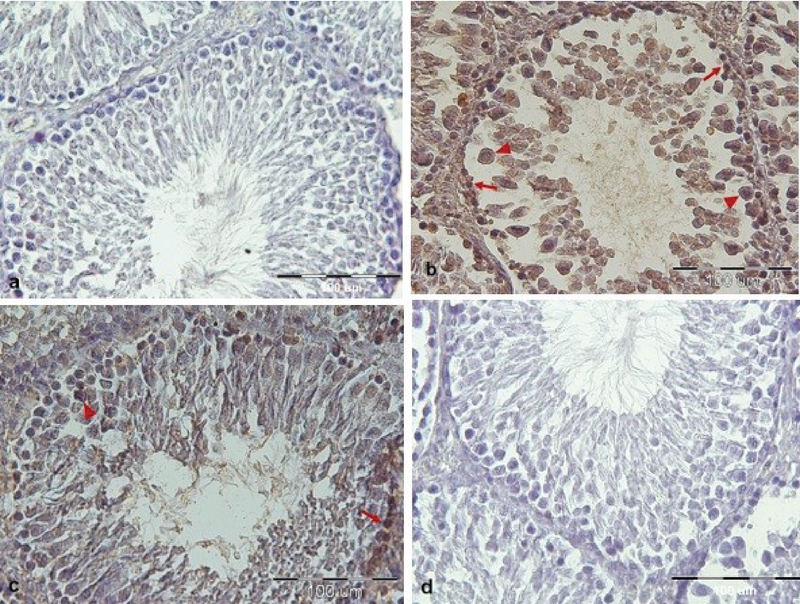
Histological sections of testicular tissues are showing TUNEL-positive spermatogonia and primary spermatocyte in different groups. (a) Control group. (b) MSG-treated group, MSG caused the increase in the apoptotic cells compared to the control group. (c) MSG co-administered with the vit C group, so that the number of apoptotic cells was decreased. (d) vit C group. Arrows show TUNEL-positive spermatogonia, arrows head show TUNEL-positive primary spermatocyte and L (lumen).

We recognized apoptotic cells in the testis of all groups by TUNEL staining. The control group contains only a few TUNEL-positive spermatogonia and primary spermatocyte. However, we saw the number and signal density of TUNEL-positive mentioned cells significantly increased in the MSG-treated group in comparison with the control group (p = 0.001). The reactivity and the number of apoptotic spermatogonia and primary spermatocyte reduced in the vit C-treatment group as compared with the MSG-treated group (p = 0.001). The apoptosis significantly decreased in the group treated with MSG co-administered with vit C as compared with the MSG-treated group (p = 0.001), and these revealed that vit C could prevent from spermatogonia and primary spermatocyte apoptosis (Figures 1 and 2).

#### Effect of MSG and vit C on germinal epithelial thickness

**Figure 3:**
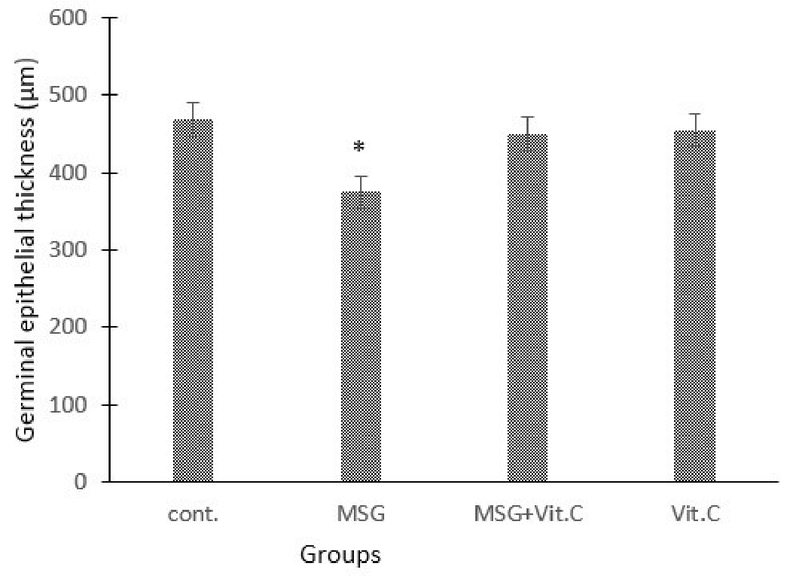
Effects of treatment of MSG, vit C, and their combinations on germinal epithelial thickness in the testis tissues of rats. Note: *represents a significant difference between MSG-treated group and control group (p < 0.05).

**Figure 4:**
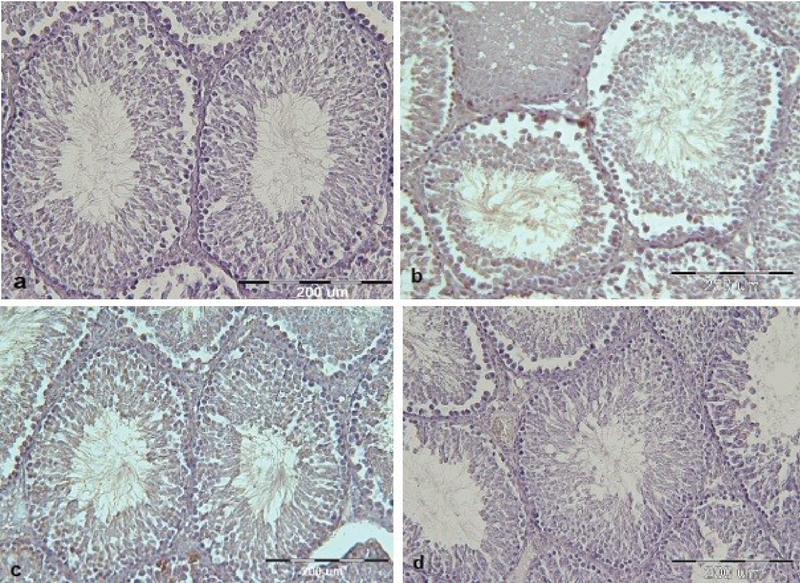
Cross-sections of testicular tissues show germinal epithelial thickness in different groups. (a) Control group. (b) MSG-treated group. (c) MSG co-administered with vit C group. (d) Vit C group. Germinal epithelial thickness shows a decrease in the MSG-treated group in comparison with other groups. Disorganization and distortion epithelial of the base membrane are seen in the MSG-treated group.

Germinal epithelial thickness significantly decreased in the MSG-treated group as compared with the control group (p = 0.036). Also, the germinal epithelial thickness in the MSG co-administered with vit C group increased as compared with the MSG group but not significantly (Figures 3 and 4).

**Table 1:** Effects of MSG and vit C on initial body weight, final body weight, weight changes, and testes' weight*


**Groups Parameter**	**Control**	**MSG**	**MSG + Vit C**	**Vit C**
Initial Body Weight (gr)	261.50 ± 5.357	229.50 ± 4.593${}^{a}$	237.83 ± 2.858${}^{ab}$	247.83 ± 3.545${}^{c}$
Final Body Weight (gr)	343.00 ± 10.75	282.50 ± 19.31${}^{a}$	298.67 ± 17.20${}^{a}$	326.00 ± 18.96${}^{b}$
Weight changes (%)	31.417	23.144${}^{a}$	25.738	31.983${}^{b}$
Testes' weight (gr)	1.50 ± 0.14	1.40 ± 0.12	1.55 ± 0.10	1.50 ± 0.06
white<bcol>5</ecol>Note: *Values are the mean ± standard deviation of the mean; ${}^{a}$Significantly different from control group (p < 0.05); ${}^{b}$Significantly different from MSG group (p < 0.05); ${}^{c}$Significantly different from MSG + vit C group (p < 0.05)
white<bcol>5</ecol>MSG: Monosodium glutamate; Vit C: Vitamin C

**Table 2:** Effects of MSG and vit C on biochemical factors (MDA, GSH) *


**Groups Parameter**	**Control**	**MSG**	**MSG + Vit**	**Vit C**
MDA (nmol/gr)	3.04 ± 0.80	3.95 ± 1.24	1.37 ± 0.77 ${}^{ab}$	2.94 ± 1.19
GSH (mmol/gr)	0.71 ± 0.03	0.62 ± 0.04	0.69 ± 0.06	0.71 ± 0.09
white<bcol>5</ecol>Note: *Values are mean ± standard deviation of the mean; ${}^{a}$Significantly different from control group (p < 0.05); ${}^{b}$Significantly different from MSG group (p < 0.05);
white<bcol>5</ecol>MSG: Monosodium glutamate; Vit C: Vitamin C; MDA: malondialdehyde; GSH: glutathione

## 4. Discussion

MSG is a common ingredient used in processed foods to enhance favor. In recent decades, MSG has been the target of studies to determine whether it is harmful to the human body. Following the absorption of MSG, glutamate receptors, α-ketoglutarate dehydrogenase and cystine-glutamate antiporter are the main causes of damage in different tissues (1, 6). Studies have found that the metabolism of high levels of glutamate can cause increased lipid peroxidation and decreased levels of major antioxidant enzymes (2, 4).

In the present study, the study group (MSG group) was compared to the control group. The biochemical parameters (MDA and GSH) in the study group changed after the use of MSG, although it was not significant, However, many studies have shown that MSG leads to the production of free radicals and related reactive oxygen species. Cellular lesions are induced by reactive oxygen species reaction with proteins and lipids, which is harmful to the human body. Furthermore, the use of MSG has been linked to metabolic disturbance and oxidative damage of tissues, which could explain the pathophysiology of many disorders or illnesses (5–7). In the present study, a low dose of 3 g/kg of MSG did not cause oxidative stress, and subsequently did not affect the activity of enzymatic antioxidants and lipid peroxidation. However, more studies should be done on antioxidant enzymes. It should be noted that MSG affects tissues through various mechanisms and oxidative stress is one of its harmful effects.

Our observation has shown that the BW of the rats increased in all groups, although the increase was lower in the MSG group than in the control group. Kondoh and his colleague found that the animals that consumed MSG were reported to have significantly less weight gain and reduced fat deposition (fat mass) and plasma leptin levels compared to the rats that consumed only water, and they linked it to GLU-sensing mechanisms (16). Also, the weight of the testicles in the MSG group was not significant compared to the control group, Ochiogu and colleagues reported that the absence of change in testis weight was probably because mature animals (adult rats) were used in the study (17).

In agreement with this result, Iamsaard and colleagues showed that the oral administration of MSG did not affect testicular weight (12). Ekaluo and colleagues and Sakr and colleagues reported that MSG-treatment caused reduction of testes' weight, epididymis weight, sperm count, and germ cell height (18, 19).

In our study also, the height of epithelial germinal decreased in the MSG group. The degeneration of seminiferous tubules and their germinal epithelium detected in this study may be due to apoptosis caused by MSG. This agrees with the results of studies that imputed pathologies on other tissue to apoptosis after MSG consumption (20, 21).

Many studies have shown the existence of glutamate system including metabotropic (mGlu) and ionotropic glutamate receptors and transporters in different tissues (22, 23). Storto and colleagues showed that mGlu5 and mGlu1 receptors are expressed in rat and human testes or human sperm (22). Activation of mGlu5 receptors has the possibility to produce intracellular Ca${}^{2+}$ waves in cells, which activates many of the reactions that play a basic role in permanence, differentiation, and cell growth (23–25). Therefore, the presence of excess glutamate because of the consumption of monosodium causes severe activation of the glutamate receptors. On the other hand, when Ca${}^{2+}$ in the cell increases or high amounts of calcium enter the organelles such as the endoplasmic reticulum, nucleus, and mitochondria, calcium-dependent enzymes, such as proteases and endonucleases (caspases) become active and provide preliminaries for apoptosis (26, 27). Hence, apoptosis occurs, which is another mechanism of MSG in testicular tissues.

MSG absorption has mostly neurotoxic effects in the brain and it affects the function of the hypothalamic-pituitary-gonadal axis. Therefore, following monosodium consumption, the testis is directly affected by glutamate receptors and can degenerate through the hypothalamus-pituitary-gonadal axis (25). Because of the effect of monosodium on the central nervous system, this axis and subsequently the levels of testosterone, follicle–stimulating hormone and luteinizing hormone, also change, causing a change in spermatogenesis.

When damage occurs, by using enzymatic and non-enzymatic antioxidants that are present in the cells, the damage is repaired, although it may not be fully completed. This study showed that ascorbic acid (vit C) was effective in ameliorating the effect of MSG in apoptotic cells. Pavlovic and colleagues showed that vit C decreased apoptosis in rat thymocytes by increasing the quantity of viable cells and up-regulating the expression of Bcl-2 protein. Hence, the presence of Bcl-2 is essential in the antioxidant defense against apoptosis. Vit C has an important role in the free radical scavengers and protection of cells as an antioxidant and participates in the synthesis of leukotrienes and collagen (28). Reducing the level of vit C in the testicular tissue and subsequently causing cell damage can be considered as another mechanism of MSG effect (29).

## 5. Conclusion 

Considering the importance of the issue of reproduction and its dependence on the lifestyle and according to our study results, vit C as an antioxidant can improve pathological and biochemical changes in testis, but not always. Hence, it may be better to reduce the use of monosodium in the foods or to remove it completely.

##  Conflict of Interest

There is no conflict of interests
